# A Distance Detector with a Strip Magnetic MOSFET and Readout Circuit

**DOI:** 10.3390/s17010126

**Published:** 2017-01-10

**Authors:** Guo-Ming Sung, Wen-Sheng Lin, Hsing-Kuang Wang

**Affiliations:** Department of Electrical Engineering, National Taipei University of Technology, Taipei 10608, Taiwan; wensheng.0@gmail.com (W.-S.L.); krs@ms15.hinet.net (H.-K.W.)

**Keywords:** distance detector, Hall effect, strip MAGFET, polysilicon cross-shaped Hall plate, magnetic sensor, readout circuit, difference amplifier, instrumentation amplifier

## Abstract

This paper presents a distance detector composed of two separated metal-oxide semiconductor field-effect transistors (MOSFETs), a differential polysilicon cross-shaped Hall plate (CSHP), and a readout circuit. The distance detector was fabricated using 0.18 μm 1P6M Complementary Metal-Oxide Semiconductor (CMOS) technology to sense the magnetic induction perpendicular to the chip surface. The differential polysilicon CSHP enabled the magnetic device to not only increase the magnetosensitivity but also eliminate the offset voltage generated because of device mismatch and Lorentz force. Two MOSFETs generated two drain currents with a quadratic function of the differential Hall voltages at CSHP. A readout circuit—composed of a current-to-voltage converter, a low-pass filter, and a difference amplifier—was designed to amplify the current difference between two drains of MOSFETs. Measurements revealed that the electrostatic discharge (ESD) could be eliminated from the distance sensor by grounding it to earth; however, the sensor could be desensitized by ESD in the absence of grounding. The magnetic influence can be ignored if the magnetic body (human) stays far from the magnetic sensor, and the measuring system is grounded to earth by using the ESD wrist strap (Strap E-GND). Both ‘no grounding’ and ‘grounding to power supply’ conditions were unsuitable for measuring the induced Hall voltage.

## 1. Introduction

The Hall plate is a major magnetic sensor, which is fabricated using CMOS technology, for sensing the magnetic induction perpendicular to the Hall plate surface and converting it into a corresponding electrical signals such as voltage, current, and frequency [1,2,3]. To enhance the linear characteristics of a current-mode Hall sensor, various compensated techniques have been developed [4,5,6]. The Hall plate performs with low-voltage-related magnetosensitivity compared with a magnetotransistor or magnetic MOSFET (MAGFET) [7]. A split-drain MAGFET is widely used as a Hall sensor for sensing the magnetic induction perpendicular to the MAGFET plane, in which a current difference is obtained between two adjacent drains of the MAGFET [8]. The relative sensitivity depends on the primary geometric parameters and biasing conditions of the applied magnetic device [9,10]. A symmetrical and differential structure performs with effective sensitivity [8]. In addition, the strip approach is considered to transmit a biasing current of up to 500 mA, whereas in the coil approach, the biasing current is limited to 20 mA [11]. The strip MAGFET with two sources is superior to that with a shared source in terms of the biasing current. In the present study, CSHP was used to detect the applied magnetic induction and to induce the differential Hall voltages, which was connected to two gates of two separated MOSFETs and generated two separated drain currents. An output voltage was obtained using a readout circuit. Notably, the magnetosensitivity was enhanced because the drain current of the MOSFET was a quadratic equation of the induced Hall voltage, which was equal to the gate voltage of the MOSFET, and the offset voltage will be effectively eliminated by the differential topology of the CSHP [12].

Magnets and magnetic induction sensing devices are often attractive in instrumentation because they can be operated in noncontacting conditions and are small in size [13]. However, the magnetic induction decays with distance according to the power–law relationship. A magnetic induction sensing device with a high sensitivity to small magnetic induction and linear response to human motion should be selected [13]. Notably, the magnetic induction strength is in the order of tens of Gauss. The proposed device in [13] could be useful in situations that require noncontacting displacement measurements. In addition, a linear bidirectional moving-magnet actuator with elastic magnetic force was presented in [14], which is an arrangement of two linear radiometric Hall sensors and a small permanent magnet disc attached to the mobile actuator’s rod that moves between the two fixed sensors. An open-loop method, proposed by Arcire [14], uses an analog flux density transducer based on the Hall effect with a microcontroller board for acquiring and processing the analog data delivered by the transducer. The determination of the displacement around a neutral equilibrium position is easy. Moreover, Netzer described two linear methods for noncontacting position measurement based on a relative movement between a Hall-element magnetic sensor and contact gradient static magnetic induction [15]. The induction intensity at the edges of the linear range was approximately ±300 G, and the maximum error due to the earth’s magnetic induction of ±0.3 G was approximately 0.1%. In addition, a new position detecting system by using the magnetic flux of the magnet on the mover was proposed, in which a Hall element was considered in a flux sensor for a synchronous motor [16]. Moreover, an intelligent odometer was studied using the Hall effect to avoid the mechanical odometer defect. The speed and mileage were measured using the Hall sensor through the pulse detection method [17].

For distance detection, an unconventional method was proposed to identify the lane markings on a road surface through laser measurement system. This method was achieved using the reflection of the laser beam [18]. Fall detection in elderly residents was performed using an infrared distance sensor [19]. Furthermore, the Poisson distance image was acquired by solving the two-dimensional Poisson equations defined on the spatial–temporal accumulative image, and the action descriptors were fed into the nearest neighbor classifier to recognize daily actions, particularly to detect the abnormal fall action [20]. In addition, an advanced driver assistance was presented to complete the measurement of the distance between a vehicle and the detected object. Particular attention has been given to the vision-based technique because it is considered one of the most accurate systems for identifying targets and measuring the distance from the target to the vehicle [21]. In brief, distance measurement can be completed using techniques such as laser beam, infrared sensor, visual-based fall detection, and vision-based technique. However, both laser beam and infrared sensor are oriental sensors, and the visual-based fall detection and vision-based technique operate in a bright environment. The object to be measured cannot be detected if it is located in the dark or has diverged from the oriental range even when it is considerably closer to the sensor. To resolve these faults, the current study proposes a distance detecting method by using the strip MAGFET, which includes two separated MOSFETs, a differential polysilicon CSHP, and a readout circuit.

The proposed distance detector could detect the distance from the magnetic body (human) to the magnetic sensor by sensing the magnetic induction of the magnetic body perpendicular to the chip surface. The strip MAGFET not only increased the magnetosensitivity but also eliminated the offset voltage which is generated because of the device mismatch and Lorentz force [22]. Moreover, a commercial instrumentation amplifier (IA) INA333 was applied to amplify the output voltage of the readout circuit and increase the detecting distance. The remainder of this paper is organized as follows. Section 2 describes the operational principles of the proposed distance detector. Section 3 presents the readout circuit for the distance detector. Section 4 presents some simulated and measured results. Section 5 presents discussion followed by conclusions.

## 2. Operational Principles of the Distance Detector

**Figure 1:**
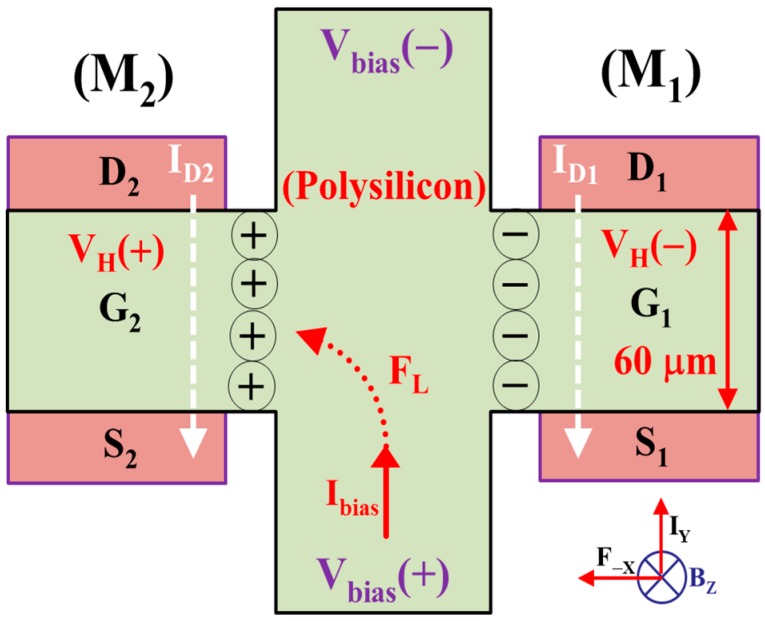
Strip MAGFET with two separated MOSFETs and a differential polysilicon CSHP. The Lorentz force F_L_ not only pushes the positive charge ⊕ to the left to gather at the left side of the polysilicon CSHP, but also injects the electron current ⊖ into the right side of the polysilicon CSHP to reduce the drain current of the first MOSFET (*I_D_*_1_).

Figure 1 presents a strip MAGFET, which is composed of two separated MOSFETs, M1 and M2, and a differential polysilicon CSHP, for sensing the magnetic induction *B_Z_* perpendicular to the chip surface. The distance from the magnetic body (human) to the magnetic device (strip MAGFET) can be easily detected using the proposed magnetic sensor. *I_bias_* is the biasing current in the *y*-direction and *F_−X_* is the Lorentz force in the *x*-direction, reversely. The differential topology not only effectively cancels the offset but also doubly enhances the magnetosensitivity. A polysilicon CSHP is located on two identical MOSFETs to establish a strip MAGFET with high biasing current [8]. In the absence of the applied magnetic induction *B_Z_*, two drain currents, *I_D_*_1_ and *I_D_*_2_, are the same at D_1_ and D_2_ of the MOSFETs, M_1_ and M_2_, respectively. After passing through the readout circuit, which includes a current-to-voltage (I-to-V) converter, a low-pass filter (LPF), and a difference amplifier (DA), the output voltage *V_OUT_* of the DA is constant. That is, ∆*V_OUT_* = 0. By contrast, the output voltage *V_OUT_* is a function of the bias current versus applied magnetic induction.

In general, the voltage-mode Hall devices can be biased in two modes: voltage biasing and current biasing. In the current biasing mode, the current-related sensitivity *S_RI_* is calculated as follows [23]:

(1) $$S_{RI}\left(B\right)=\left|\frac{1}{I_{bias}}\frac{\Delta V_{OUT}}{\Delta B}\right|$$

where the unit of *S_RI_(B)* is V·A^−1^·T^−1^, *I_bias_* represents the supply bias current, *∆B* represents the change in the applied magnetic induction, and ∆*V_OUT_* represents a variation in the DA output voltage [23]. However, the proposed strip MAGFET detects the distance *∆D* between the magnetic sensor and magnetic body. Thus, the current-related sensitivity should be redefined as follows:

(2) $$S_{RI}\left(D\right)=\left|\frac{1}{I_{bias}}\frac{\Delta V_{OUT}}{\Delta D}\right|$$

where the unit of *S_RI_(D)* is V·A^−1^·m^−1^. In addition, the nonlinearity error (*NLE*) is defined as

(3) $$NLE=\left|\frac{\Delta V_{OUT}-\Delta V_{OUT}^{\left(0\right)}}{\Delta V_{OUT}^{\left(0\right)}}\right|\times 100\%$$

where the unit of *NLE* is % and ∆*V*^(0)^*_OUT_* represents the calculated output voltage difference based on the slope of the straight line obtained from the best fit to the output characteristic [24]. The operational principle of the strip MAGFET is described below.

As shown in Figure 1, two identical MOSFETs, M_1_ and M_2_, are operated in the saturation mode. If a magnetic induction *B_Z_* and a bias current *I_bias_* are applied to the polysilicon CSHP, the positive and negative Hall voltages, *V_H_(+)* and *V_H_(−)*, are generated by Lorentz force *F_L_* in the *x*-direction reversely and *x*-direction, respectively. In other words, the gate voltage of M_2_ is larger than that of M_1_. By grounding two sources, S_1_ and S_2_, two drain currents, *I_D_*_1_ and *I_D_*_2_, can be expressed as follows:

(4) $$\{\begin{array}{c} I_{D1}=\frac{1}{2}\mu_{n}C_{ox}\frac{W}{L}{\left(V_{GS1}-V_{TH}\right)}^{2}\\ I_{D2}=\frac{1}{2}\mu_{n}C_{ox}\frac{W}{L}{\left(V_{GS2}-V_{TH}\right)}^{2} \end{array}$$

and

(5) $$V_{GS2}-V_{GS1}=V_{H}\left(+\right)-V_{H}\left(-\right)=V_{H}$$

(6) $$\frac{V_{GS1}+V_{GS2}}{2}=V_{GS}$$

where *μ_n_*, *C_ox_*, *W*, *L*, and *V_TH_* represent the mobility of electrons, parasitic capacitance per unit gate area, width, length, and threshold voltage, respectively. *V_GS_*_1_ and *V_GS_*_2_ represent the differential gate-to-source voltages of M1 and M2, respectively, with magnetic induction, whereas *V_GS_* represents the gate-to-source voltage without magnetic induction. Subsequently, the induced Hall voltage *V_H_* is derived from the Lorentz force.

(7) $$V_{H}=G\;R_{H}I_{bias}B_{Z}/t$$

where $R_{H}=-\mu_{n}^{*}/\sigma_{n}=-r_{n}/nq$ represents the Hall coefficient, *I_bias_* represents the bias current in the *y*-direction, *B_Z_* represents the magnetic induction in the z-direction, and *t* and *G* represent the polysilicon thickness and geometrical correction factor, respectively [24]. Thus, the current difference between *I_D_*_1_ and *I_D_*_2_ can be expressed as follows:

(8) $$\Delta I_{D}=I_{D2}-I_{D1}=\frac{1}{2}\mu_{n}C_{ox}\frac{W}{L}\left[2\left(\frac{V_{GS1}+V_{GS2}}{2}-V_{TH}\right)\left(V_{GS1}-V_{GS2}\right)\right]$$

Rewriting Equation (8), we have

(9) $$\Delta I_{D}=\mu_{n}C_{ox}\frac{W}{L}\times \left(V_{GS}-V_{TH}\right)V_{H}=\mu_{n}C_{ox}\frac{W}{L}\left(\frac{GR_{H}I_{bias}B_{Z}}{t}\right)\left(V_{GS}-V_{TH}\right)$$

As shown in Equation (9), the current difference *∆I_D_* is directly proportional to the bias current *I_bias_* and the magnetic induction *B_Z_*. If the bias current *I_bias_* is set as constant, the larger the magnetic induction *B_Z_* is, the higher the drain current difference *∆I_D_* is. The physical mechanism may be that the Lorentz force *F_L_* pushes the positive charge to the left to gather at the left side of the polysilicon CSHP, which is connected to the gate terminal G_2_ of the second MOSFET (M_2_). Moreover, the electron current is directly injected into the right side of the polysilicon CSHP, which is connected to the gate node G_1_ of the first MOSFET (M_1_), to reduce the drain current of the first MOSFET (*I_D_*_1_). By applying a magnetic induction (~mT) to the magnetic sensor, an output voltage *V_OUT_* is acquired at the output node of the readout circuit by multiplying the drain current difference ∆*I_D_* with a resistor *R_D_* in the I-to-V converter and then multiplying approximately 16 times by *R_A_* = 100 kΩ and *R_B_* = 6.2 kΩ.

## 3. Readout Circuit

**Figure 2:**
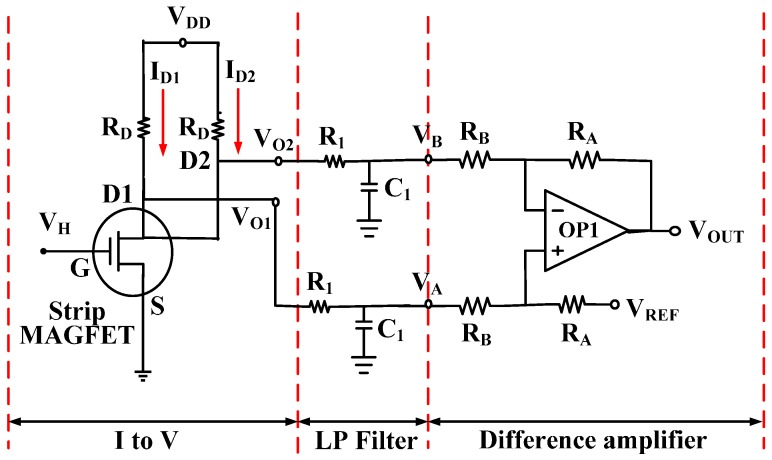
Readout circuit of the proposed distance detector comprising an I-to-V converter, low-pass filter (LPF), and difference amplifier (DA).

Two drain currents, *I_D_*_1_ and *I_D_*_2_, obtained at two separated drains of two MAGFETs over the considered magnetic induction range, are of the order of few milliamperes (mA). By passing through the I-to-V converter with resistor *R_D_* and LPF with resistor *R*_1_ and capacitor *C*_1_, the high-frequency noise will be filtered out before the entering the DA. Figure 2 presents the readout circuit of the proposed distance detector, which includes the I-to-V converter, LPF, and DA. Notably, the offset voltage can be effectively removed using the DA, and the single output *V_OUT_* obtained is easy to use.

As shown in Figure 2, an I-to-V converter was selected to convert the induced drain current, obtained from the proposed strip MAGFET, into a Hall voltage with drain resistor *R_D_*. Two output voltages, *V_O_*_1_ and *V_O_*_2_, of the I-to-V converter can be expressed as follows:

(10) $$\{\begin{array}{c} V_{O1}=V_{DD}-I_{D1}\times R_{D}\\ V_{O2}=V_{DD}-I_{D2}\times R_{D} \end{array}$$

where *V_DD_* represents the power supply. The output voltage of the DA, *V_OUT_*, passing through the LPF with resistor *R*_1_ and capacitor *C*_1_, is given by

(11) $$V_{OUT}=V_{REF}-\Delta I_{D}\times R_{D}\times \left(\frac{1}{1+sR_{1}C_{1}}\right)\times \frac{R_{A}}{R_{B}}$$

where *V_REF_* represents a constant reference voltage, which is used to adjust the output level. *∆I_D_* (= *I_D_*_1_ − *I_D_*_2_) is defined as the drain current difference between the first and second drains of the MAGFET. For a DC magnetic induction, *s* = 0, the small-signal output voltage *v_out_* is given by

(12) $$v_{out}=-\Delta I_{D}\times R_{D}\times \frac{R_{A}}{R_{B}}$$

**Figure 3:**
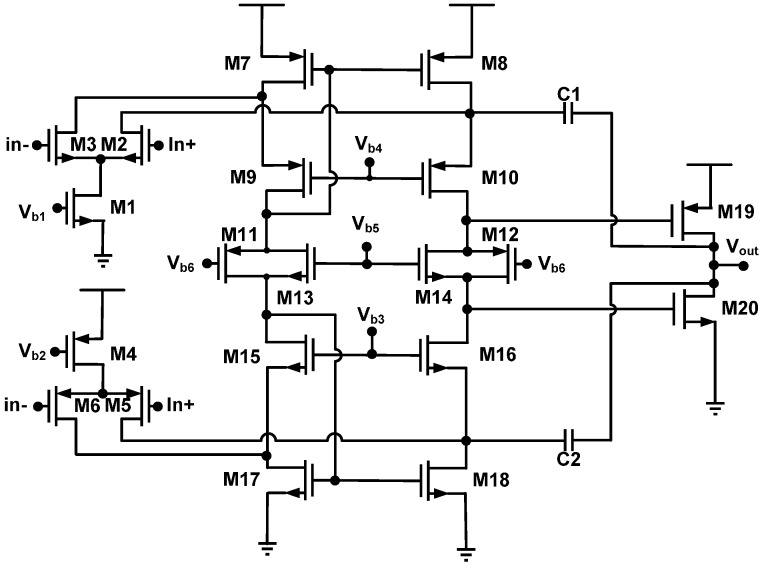
Low-voltage two-stage cascaded operational amplifier with Miller compensation and compact class-AB output stage.

Figure 3 presents a low-voltage two-stage cascaded operational amplifier (opamp) with Miller compensation and compact class-AB output stage, which is composed of two common-source-connected output transistors, M_19_ and M_20_ [25]. To make the quiescent current of the output transistors insensitive to supply voltage variations, the floating current source should have the same supply voltage dependency as the class-AB control. The value of the current source is set using two bias voltages, V_b5_ and V_b6_. The mirrors, M_7_–M_10_ and M_15_–M_18_, are loaded with the drain currents of the input pairs M_2_–M_3_ and M_5_–M_6_, respectively. These drain currents and the gate–source voltages of M7 and M17 change the common-mode input voltage. By adjusting two input bias currents with two bias voltages, V_b1_ and V_b2_, the common-mode input voltage approaches the positive supply rail or the negative supply rail. The opamp is compensated by using the conventional Miller technique including the cascode stages, M_12_ and M_14_, in the Miller loops. This compensation technique shifts the output pole to a frequency of approximately [25].

(13) $$\omega_{out}=\frac{C_{M}}{C_{GS,out}}\times \frac{g_{m0}}{C_{L}}$$

where *g_m_*_0_ represents the transconductance of the output transistors and *C_L_* represents the load capacitor. *C_M_* and *C_GS_*_,*out*_ represent the total Miller capacitor and the total gate–source capacitance of the output transistor, respectively.

**Figure 4:**
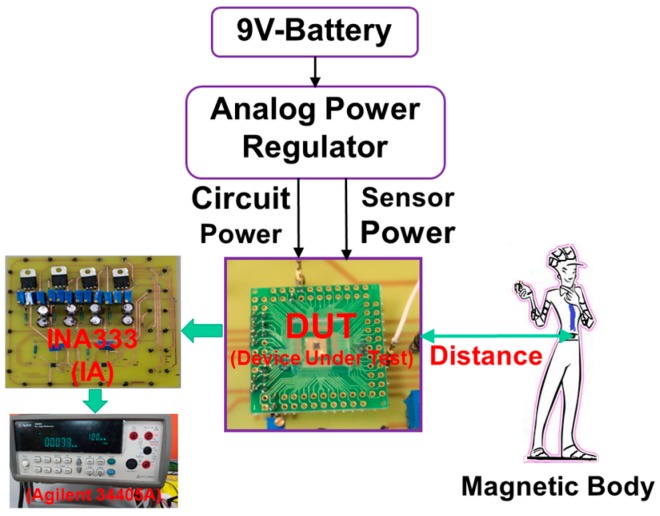
Experimental setup of distance detection.

**Figure 5:**
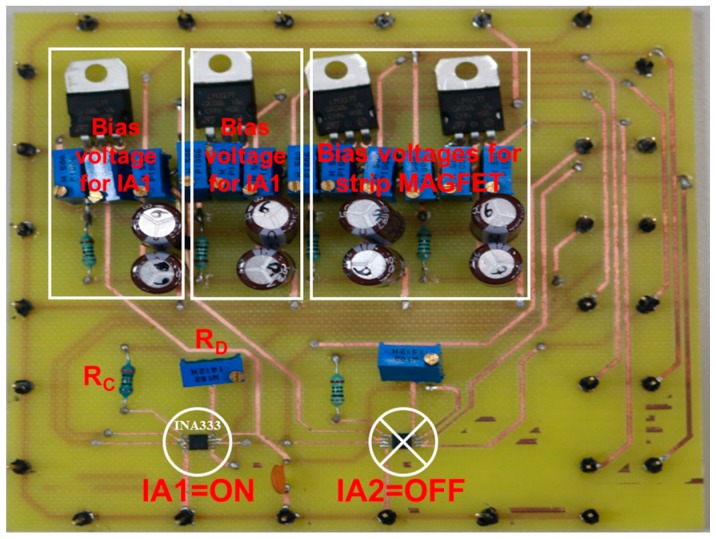
Applied PCB with two commercial instrumentation amplifiers (INA333).

Figure 4 presents the experimental setup of the distance detector composed of a device under test (DUT), an IA (INA333, Texas Instruments, Dallas, TX, USA) printed circuit board (PCB), a digital multimeter (Agilent 34405A, Santa Clara, CA, USA), a magnetic body (human), and a power regulator with a 9-V battery. The DUT included the strip MAGFET and readout circuit, and the magnetic body was actually a human weighing approximately 65 kg. The distance was defined from the chip center of the DUT to the weight center of the magnetic body. Notably, the proposed distance detecting method is not only a universal detection method but also operates in dark conditions away from light emission. To enhance the sensitivity of the distance detection and to increase the detecting distance, a commercial IA (INA333) was applied to amplify the output voltage of the readout circuit. Figure 5 presents the applied PCB that includes two commercial IAs, two bias voltages for IAs, two bias voltages for the proposed strip MAGFET, and resistors, *R_C_* and *R_D_*. By adjusting the variable resistor *R_D_*, the magnifying power of IA1 was set to 43 dB (approximately 150 times).

**Figure 6:**
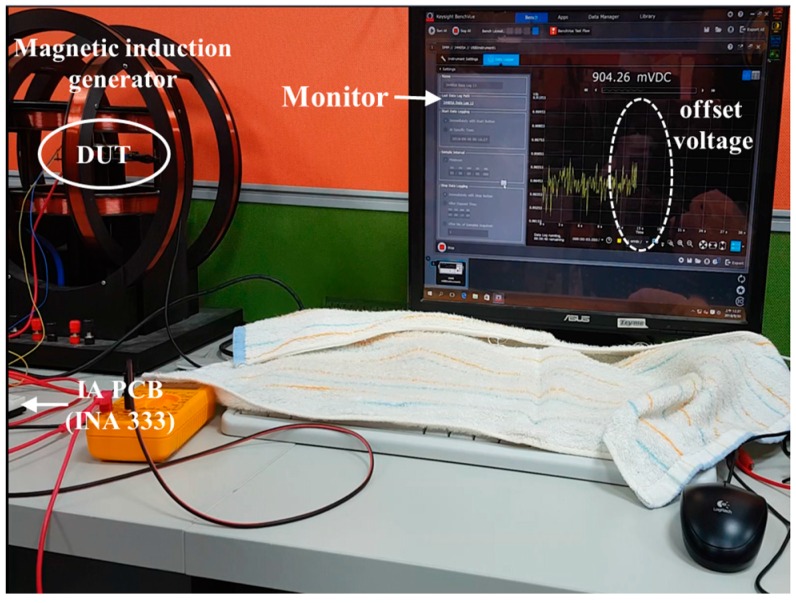
Offset voltage (right circle) displayed on the monitor without a magnetic body and DUT (Device under test) placed in the magnetic induction generator.

**Figure 7:**
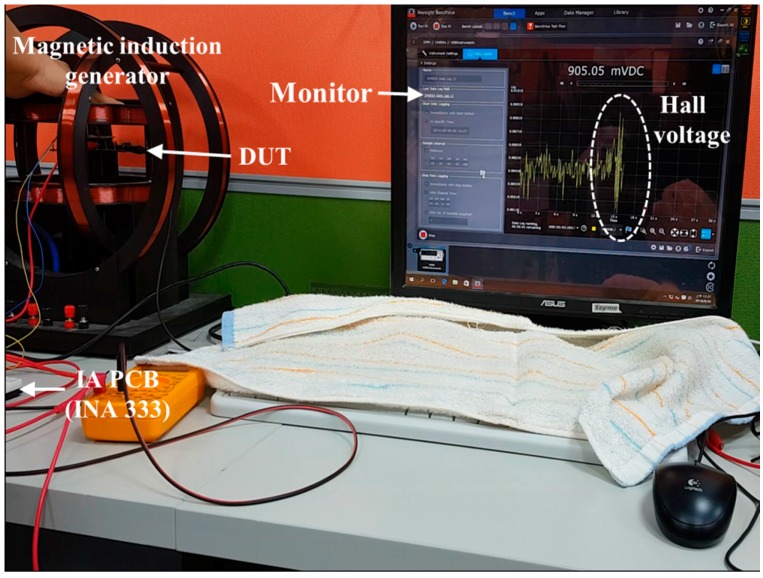
Hall voltage appears on the monitor evidently when the magnetic body comes in contact with the distance detector.

Figure 6 and Figure 7 present the measured equipment that includes a magnetic induction generator, a commercial IA (INA333) PCB, and a display monitor. As shown in Figure 6, only the offset voltage was displayed on the monitor and not the magnetic body. When the magnetic body contacts with the distance detector, Hall voltage evidently appeared on the monitor, as shown in Figure 7. A quality analysis of the measured Hall voltage is presented in Figure 7. To avoid bias, a digital multimeter (Agilent 34405A) was used for the quantity analysis of the distance detector that included a strip MAGFET, readout circuit, and IA PCB (INA333).

## 4. Simulated and Measured Results

This paper presents a low-voltage two-stage cascaded opamp with Miller compensation and compact class-AB output stage based on 0.18 μm CMOS technology for distance detection [25]. The simulated results demonstrated that the designed two-stage cascaded opamp had an open-loop gain of 75.3 dB, a phase margin of 54.9°, a common-mode rejection ratio (CMRR) of 123 dB, a slew rate of 0.07 V/μs, an input common-mode range (ICMR) of 0.9 V, a power supply rejection ratio (PSRR) of 119 dB, an output swing of 1.70 V, power consumption of 179 μW for a power supply of 1.8 volts, and a load capacitor of 10 pF.

**Figure 8:**
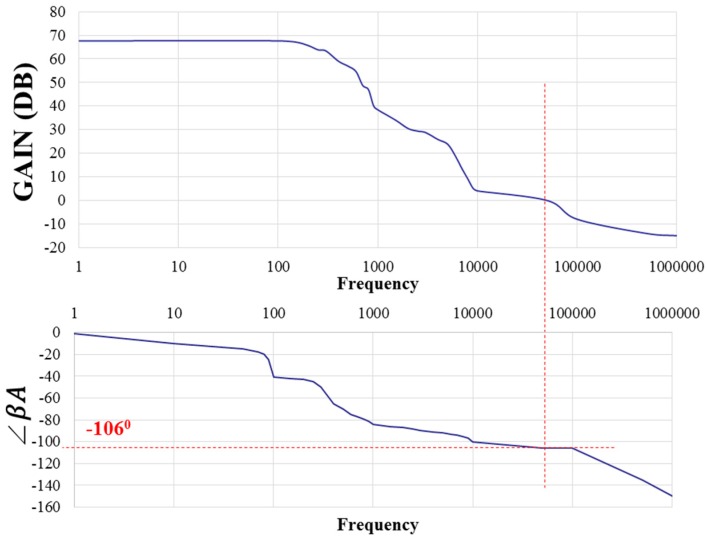
Measured open-loop gain of 67.7 dB and phase of 74° versus frequency (Hz).

**Figure 9:**
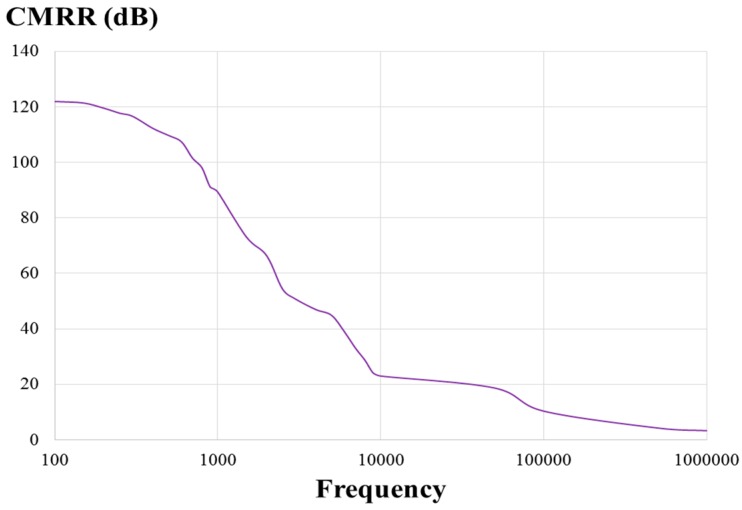
Measured common-mode rejection ratio (CMRR) of the designed opamp.

**Figure 10:**
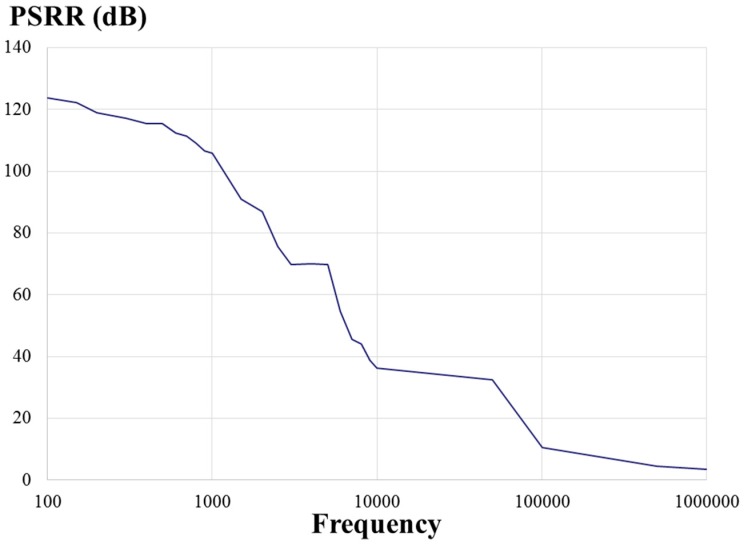
Measured power supply rejection ratio (PSRR) of the designed opamp with respect to frequency (Hz).

**Figure 11:**
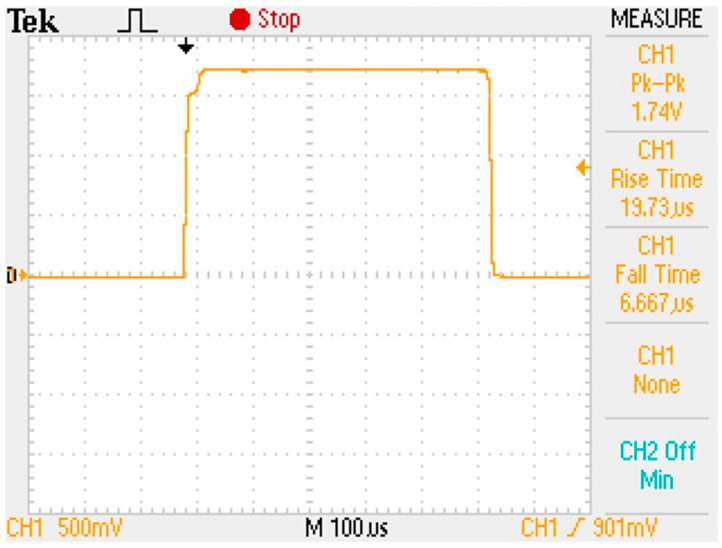
Measured slew rate of 0.07 V/μs, rise time of 19.73 μs, and fall time of 6.67 μs at an operating frequency of 1 kHz.

**Figure 12:**
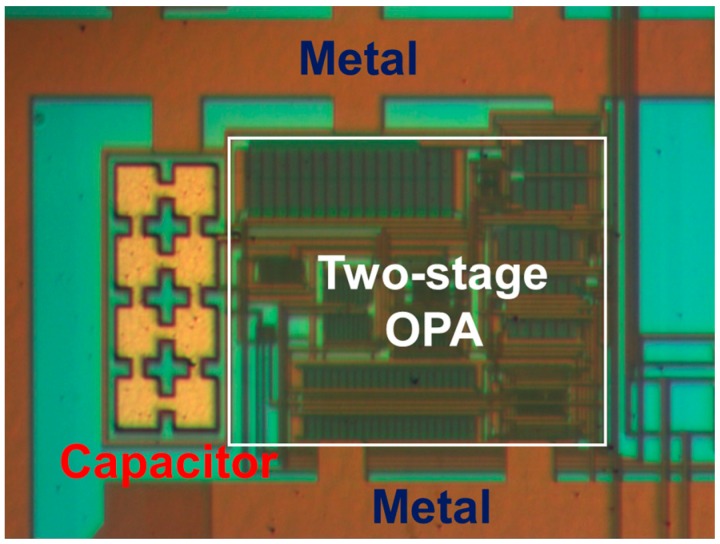
Microphotograph of the designed low-voltage two-stage cascaded opamp without external resistors and capacitors.

Figure 8 presents a measured open-loop gain of 67.7 dB and phase of 74° with respect to frequency. The measured open-loop gain was lower than that obtained through simulation, but the measured phase was higher than that obtained through simulation. Figure 9 and Figure 10 present the measured CMRR and PSRR, respectively, which were approximately equal to the relative simulated results. Furthermore, Figure 11 presents a measured slew rate of 0.07 V/μs, rise time of 19.73 μs, and fall time of 6.67 μs at an operating frequency of 1 kHz. The simulated and measured results compared with those of previous studies are tabulated in Table 1. Measured results of voltage gain, phase margin, CMRR, slew rate, ICMR, PSRR, and output swing, were more favorable than those obtained in previous studies [26,27]. Although these previous studies have not proved the efficiency, the measured data in the present study verified that the designed opamp worked as intended. Figure 12 presents the microphotograph of the designed opamp without external resistors and capacitors. Notably, an output voltage *V_OUT_* was acquired at the output node of the DA by multiplying the drain current difference ∆*I_D_* with external resistors, *R_A_* = 100 kΩ and *R_B_* = 6.2 kΩ. That is, the voltage gain of the DA was approximately 17 times greater.

To elucidate the detection mechanism, the experimental setup can be divided into three categories: direct measurement without grounding (direct), grounding to earth with electrostatic discharge (ESD) wrist strap (Strap E-GND), and grounding to power supply with ESD wrist strap (Strap S-GND). The first category elucidates the influence of ESD without grounding. Next, ESD will be eliminated from the magnetic sensor by grounding it to earth. Finally, in the third category, the ESD wrist strap is grounded to the power supply, and not to earth. This connection will enable the removal of ESD from the magnetic sensor to the power supply; however, ESD always exists between the power supply and earth.

**Figure 13:**
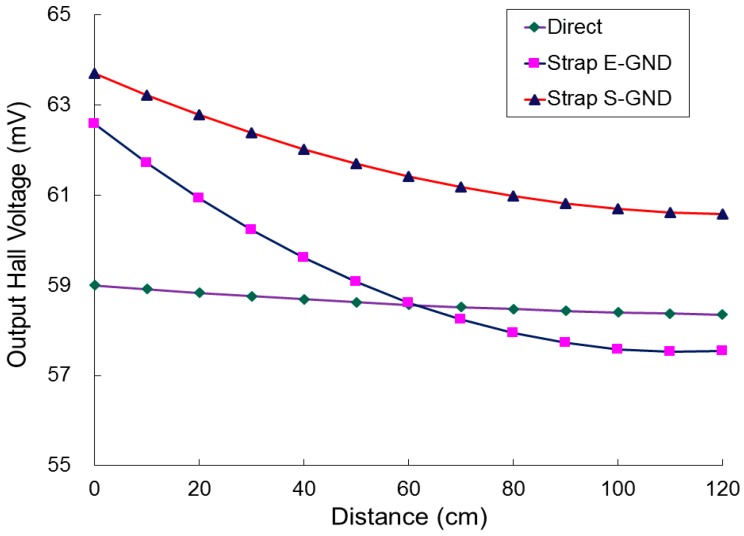
Measured output Hall voltages as a function of the distance, from 0 to 120 cm in steps of 10 cm, at a bias current of 12 mA for the proposed strip MAGFET with readout circuit and IA PCB (INA333).

Figure 13 presents the measured output Hall voltages as a function of the distance, from 0 cm to 120 cm in steps of 10 cm, at a CSHP bias current (*I_bias_*) of 12 mA. In the first category, the measured output Hall voltage varied from 58.35 to 59.00 mA. The voltage variation was approximately 0.65 mV. In the second category, the variation in the measured output Hall voltage was approximately 5.04 mV, from 57.54 to 62.58 mV. In the third category, the variation in the measured output Hall voltage was approximately 3.12 mV, from 60.58 to 63.70 mV. According to the measured Hall voltage, the second category performed with a more efficient sensitivity than the other two, and the first category performed with minimum sensitivity of nearly zero. That is, ESD could be eliminated from the magnetic sensor by grounding it to earth; however, ESD will cancel the sensitivity out when the sensor is not grounded. Notably, saturation was observed in the second category. This result indicates that the magnetic influence can be ignored if the human stayed far from the DUT, which was grounded to earth by using the ESD wrist strap (Strap E-GND). However, the first and third categories were still influenced by the magnetic body (human). Both ‘no grounding’ and ‘grounding to power supply’ were unsuitable for measuring the induced Hall voltage although the magnetic body was far and away from the designed DUT.

**Figure 14:**
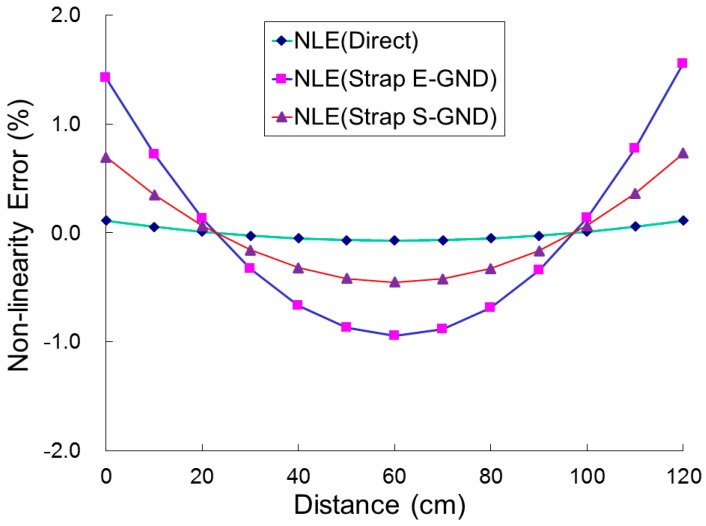
Measured nonlinearity errors (*NLE*) in categories 1, 2, and 3, against the distance, from 0 to 120 cm in steps of 10 cm, for the proposed strip MAGFET with a readout circuit and an IA PCB (INA333).

Figure 14 presents the measured *NLE* as a function of distance, from 0 to 120 cm in steps of 10 cm, and various categories for the designed strip MAGFET with a readout circuit and an IA PCB. The variation in *NLE* was large when the MAGFET was operated in category 2 conditions in which the measurement is completed by grounding the sensor to earth with an ESD wrist strap (Strap E-GND). *NLE* was nearly constant when the measurement was finished without grounding. The sensor performed with a linear characteristic when operated in category 1, but yielded a quadratic curve in categories 2 and 3. Notably, *NLE* was equal to zero at two distances, 20 and 100 cm. The measured output voltage difference ∆*V_OUT_* was equal to the calculated output voltage difference ∆*V*^(0)^*_OUT_* on the basis of the slope of the straight line obtained from the best fit to the output characteristic [24].

**Figure 15:**
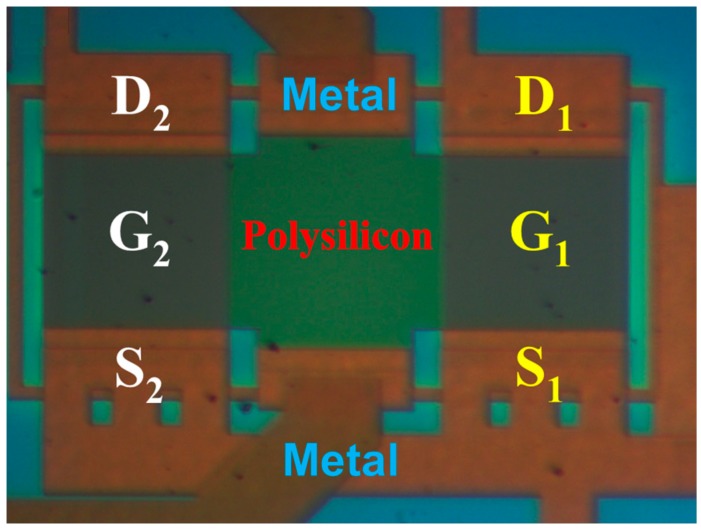
Microphotograph of the proposed strip MAGFET comprising two separated MOSFETs and a differential polysilicon cross-shaped Hall plate (CSHP).

Table 2 summarizes the measured results of the proposed strip MAGFET with a readout circuit and an IA PCB (INA333). The average nonlinearity *NLE*_ave_ and the maximum nonlinearity *NLE*_max_ were proportional to the measured Hall voltage difference ∆*V_H_* and the current-related sensitivity *S_RI_*(*D*) with a bias current of 12 mA. The maximum current-related magnetosensitivity was 0.35 V/A·m at an output Hall voltage of 5.04 mV and a distance of 0 cm (i.e., the magnetic body contacts with DUT). The measured results demonstrated that the proposed strip MAGFET was an effective distance sensor; however, it presented poor linearity. Figure 15 presents a microphotograph of the proposed strip MAGFET with a chip area of approximately 100 × 220 μm^2^.

## 5. Conclusions

In this paper, a strip MAGFET with a readout circuit and an IA PCB (INA333) is presented to measure the distance from a magnetic body (human) to the DUT, which was fabricated using 0.18 μm 1P6M CMOS technology. The object to be measured cannot be detected using the proposed magnetic sensor if the object is located in the dark or has diverged from the oriental range even when the object is considerably closer to the sensor. The physical mechanism underlying this phenomenon is that the Lorentz force pushes the positive charge to the left to gather at the left side of the polysilicon CSHP, which is connected to the gate terminal of the second MOSFET (M_2_). The current difference ∆*I_D_* between the two drains will be amplified linearly. After passing through the readout circuit, the magnetosensitivity of the sensor will be improved effectively by amplifying the current difference ∆*I_D_*. The experimental results revealed that ESD could be eliminated from the magnetic sensor by grounding the sensor to earth; however, the magnetosensitivity is canceled out without grounding. Furthermore, the magnetic influence can be ignored if the human stays far from the DUT, which is grounded to earth by using the ESD wrist strap (Strap E-GND). Both ‘no grounding’ and ‘grounding to power supply’ were unsuitable for measuring the induced Hall voltage of the magnetic body. Notably, the *NLE* was nearly constant when the measurement was completed without grounding. The sensor performed with a linear characteristic in category 1, whereas yielded a quadratic curve in categories 2 and 3. The maximum current-related magnetosensitivity of 0.35 V/A·m was obtained at an output Hall voltage of 5.04 mV and a distance of 0 cm (i.e., the magnetic body was in contact with the DUT). According to the measured results, the proposed strip MAGFET with a readout circuit and an IA PCB (INA333) is an efficient distance detector. Moreover, this study is unique because it compensates for the lack of the research literature in Open Access Publishing related to distance detection by using a magnetic sensor.

## Figures and Tables

**Table 1:** Comparison of simulated and measured results of amplifier with results of previous designs.

Specifications	This Work		[26]	[27]
	Simulation	Measurement	Simulation	Simulation
Technology	0.18 μm	0.18 μm	0.18 μm	0.5 μm
Voltage gain (dB)	75.3	67.7	67.7	45
Phase margin (°)	54.9	74	-	-
CMRR (dB)	123	122	92	75
Slew rate (V/μs)	0.07	0.07	-	-
ICMR (V_PP_)	0.9	0.744	-	-
Gain bandwidth (kHz)	1.85	0.225	1.75	5.8
UGB (MHz)	7.85	-	91.33	-
PSRR(+) (dB)	119	123	-	-
Output swing (V)	1.70	1.32	-	-
Power dissipation (μW)	179	274	263	280
Chip area (mm^2^)	0.0153	0.0153	1.49	-

**Table 2:** Measurements of maximum output Hall voltages.

Category	∆*V_H_* (mV)	*S_RI_*(*D*) (V/A·M)	*NLE*_ave_ (%)	*NLE*_max_ (%)	*I_bias_* (mA)	Distance (cm)	Grounded Mode
1	0.65	0.045	0.055	0.112	12	120	Direct
2	5.04	0.350	0.729	1.426	12	120	Strap E-GND
3	3.12	0.217	0.349	0.732	12	120	Strap S-GND

**∆***V_H_*; optimum current-related magnetosensitivities, *S_RI_*(*D*); average nonlinearity error, *NLE*_ave_; maximum nonlinearity error, *NLE*_max_; and maximum distance at a bias current of 12 mA for the proposed strip MAGFET with a readout circuit and an IA PCB.
